# Supplementary material for: Alterations of the Gut Microbiota and Metabolomics Associated with the Different Growth Performances of Macrobrachium rosenbergii Families
Source: Animals (Basel). 2023 May 4;13(9):1539. doi: 10.3390/ani13091539 (PMC10177557; doi:10.3390/ani13091539)
Supplement: Supplementary file 1 [file animals-13-01539-s001.zip › Figure S1.pdf]

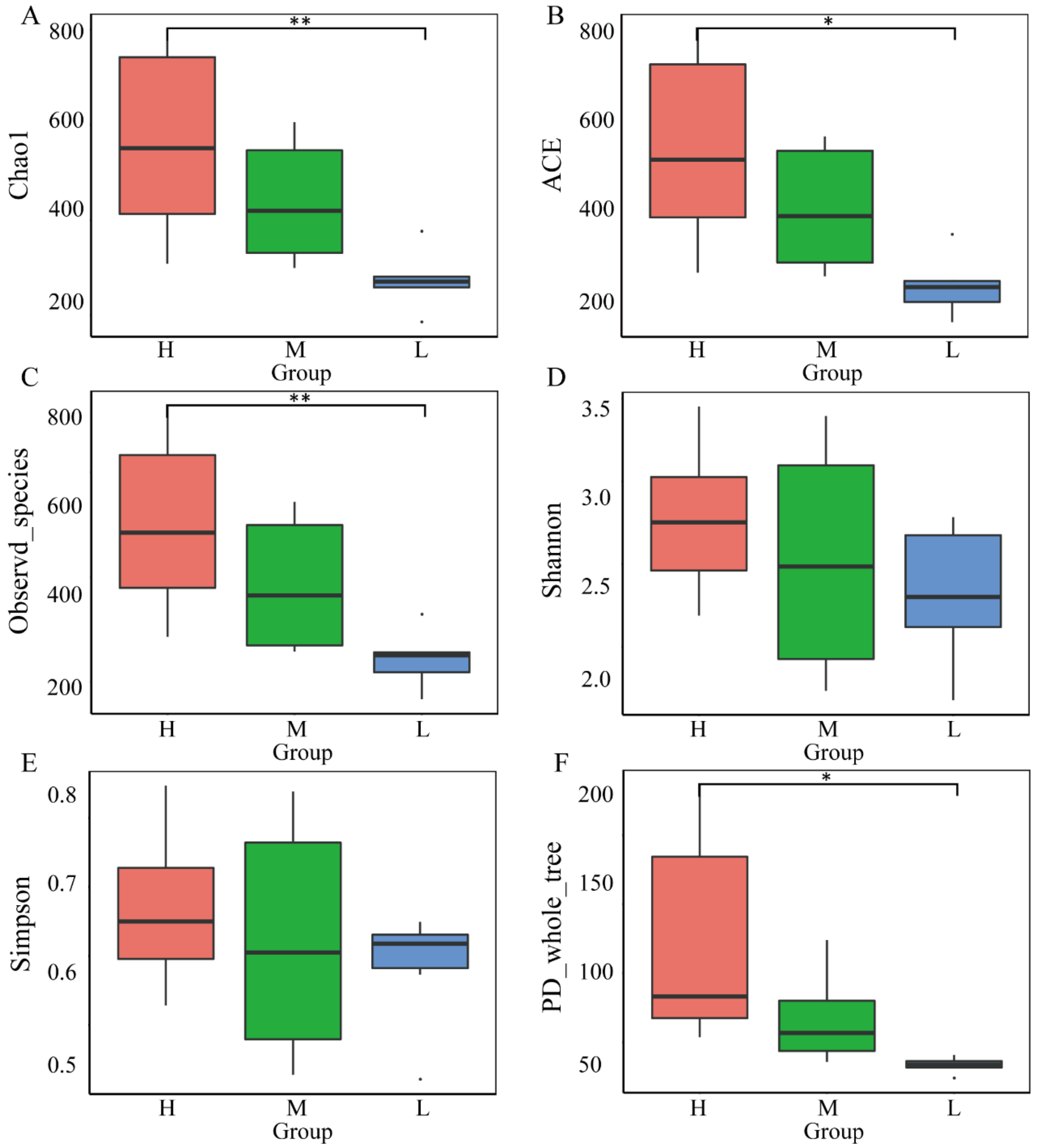

Figure S1.

Box plots of alpha-diversity show differences among the three groups. (A) Chao1 index. (B) ACE index. (C) Observed species index. (D) Shannon index. (E) Simpson index. (F) PD whole tree index. The significance of any difference among the three groups is determined by Tukey's test, * indicates significant (p<0.05), ** indicates highly significant (p<0.01). H, high growth performance level; M, medium growth performance level; L, low growth performance level.
