# Supplementary material for: Alterations of the Gut Microbiota and Metabolomics Associated with the Different Growth Performances of Macrobrachium rosenbergii Families
Source: Animals (Basel). 2023 May 4;13(9):1539. doi: 10.3390/ani13091539 (PMC10177557; doi:10.3390/ani13091539)
Supplement: Supplementary file 1 [file animals-13-01539-s001.zip › Figure S2.pdf]

A

B

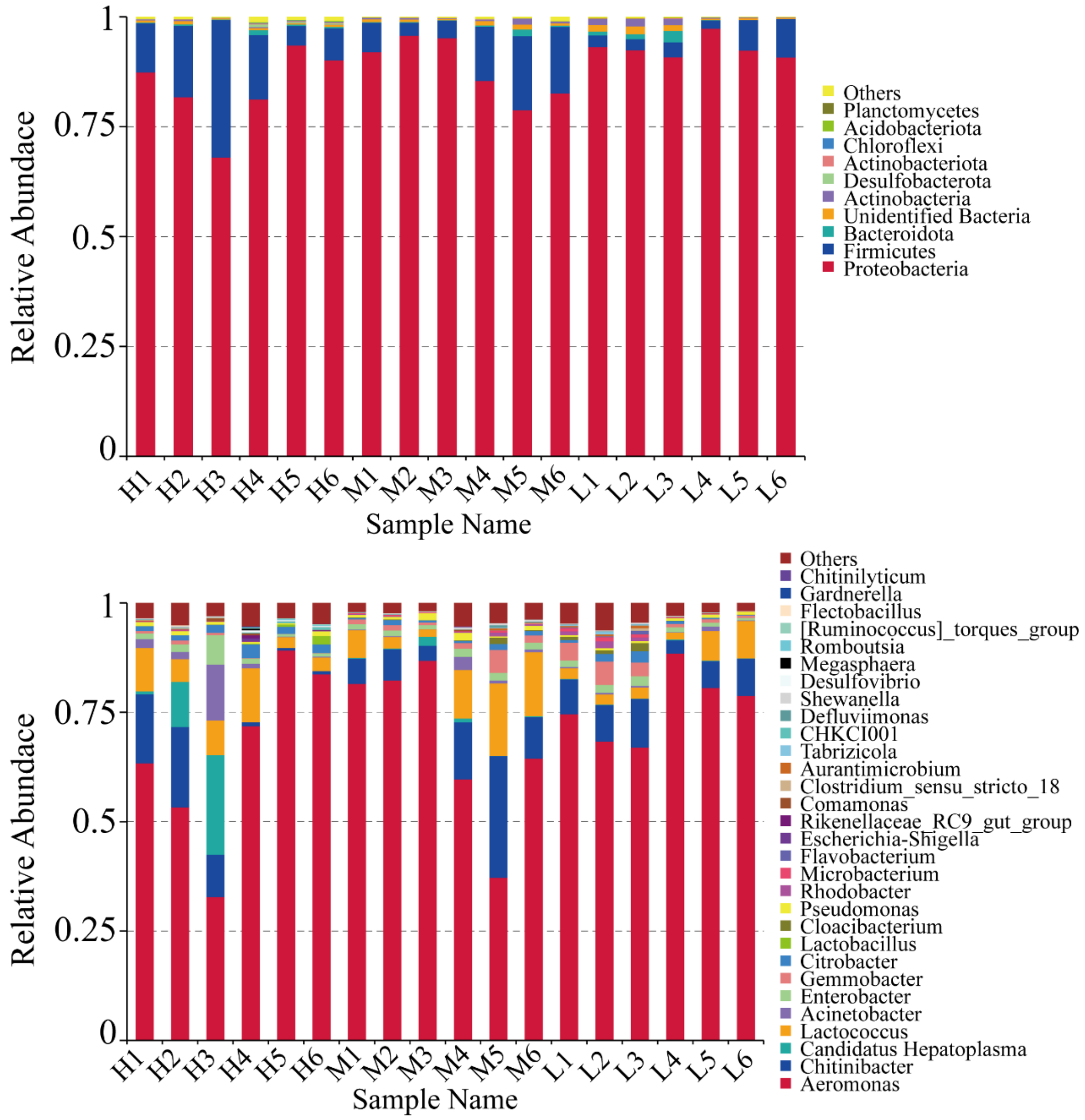

Figure S2.

The relative abundance of gut microbiota of GFP families with three growth performance groups at TOP10 of phylum (A) and TOP30 of genus (B) levels. H, high growth performance level; M, medium growth performance level; L, low growth performance level.
