# Supplementary material for: Alterations of the Gut Microbiota and Metabolomics Associated with the Different Growth Performances of Macrobrachium rosenbergii Families
Source: Animals (Basel). 2023 May 4;13(9):1539. doi: 10.3390/ani13091539 (PMC10177557; doi:10.3390/ani13091539)
Supplement: Supplementary file 1 [file animals-13-01539-s001.zip › Figure S4.pdf]

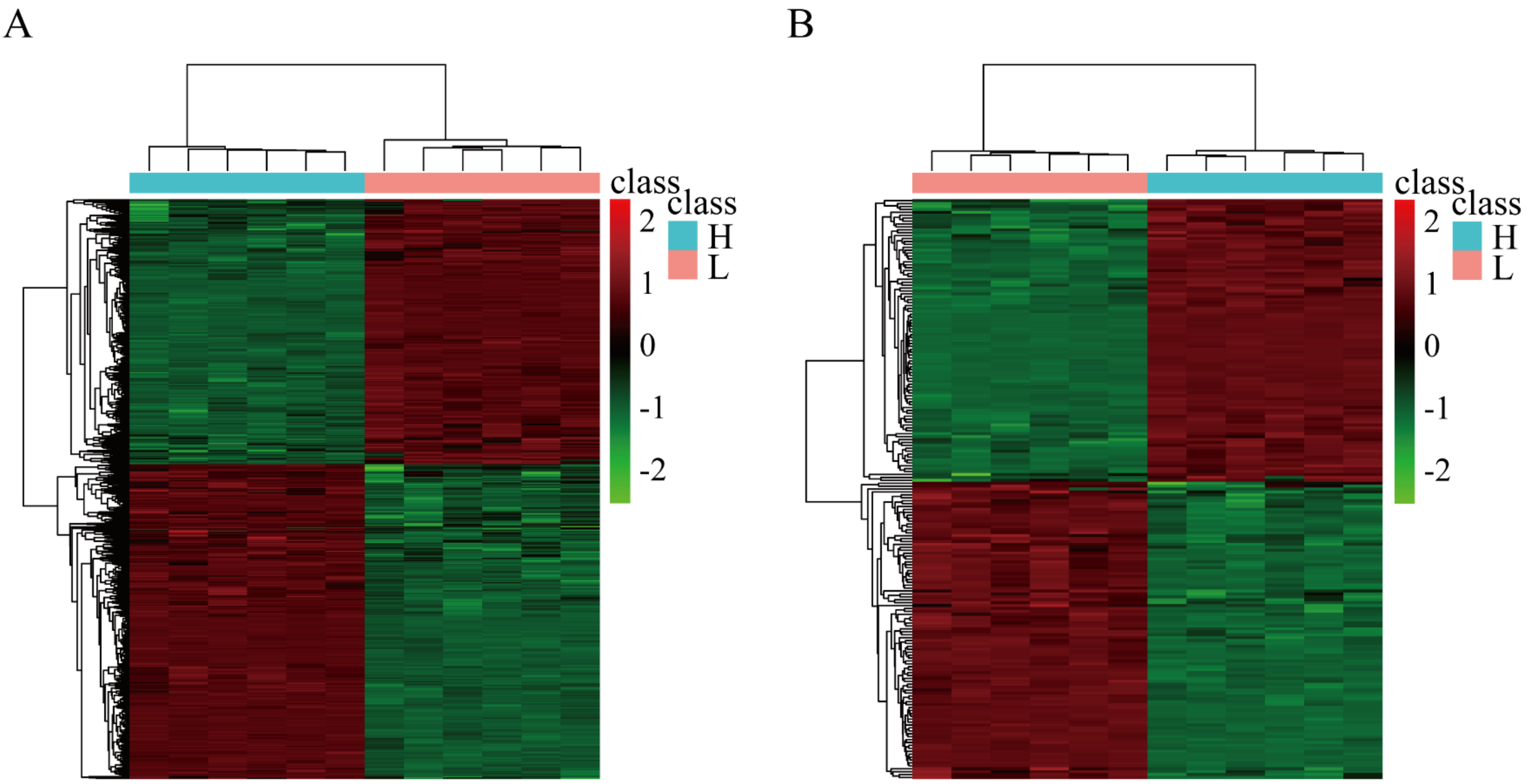

Figure S4:

Heatmaps of the differential abundance of metabolites in ESI$^+$ (A) and ESI$^-$ (B) scan modes between H and L groups. H, high growth performance level; L, low growth performance level.
