# Supplementary material for: Alterations of the Gut Microbiota and Metabolomics Associated with the Different Growth Performances of Macrobrachium rosenbergii Families
Source: Animals (Basel). 2023 May 4;13(9):1539. doi: 10.3390/ani13091539 (PMC10177557; doi:10.3390/ani13091539)
Supplement: Supplementary file 1 [file animals-13-01539-s001.zip › Figure S5.pdf]

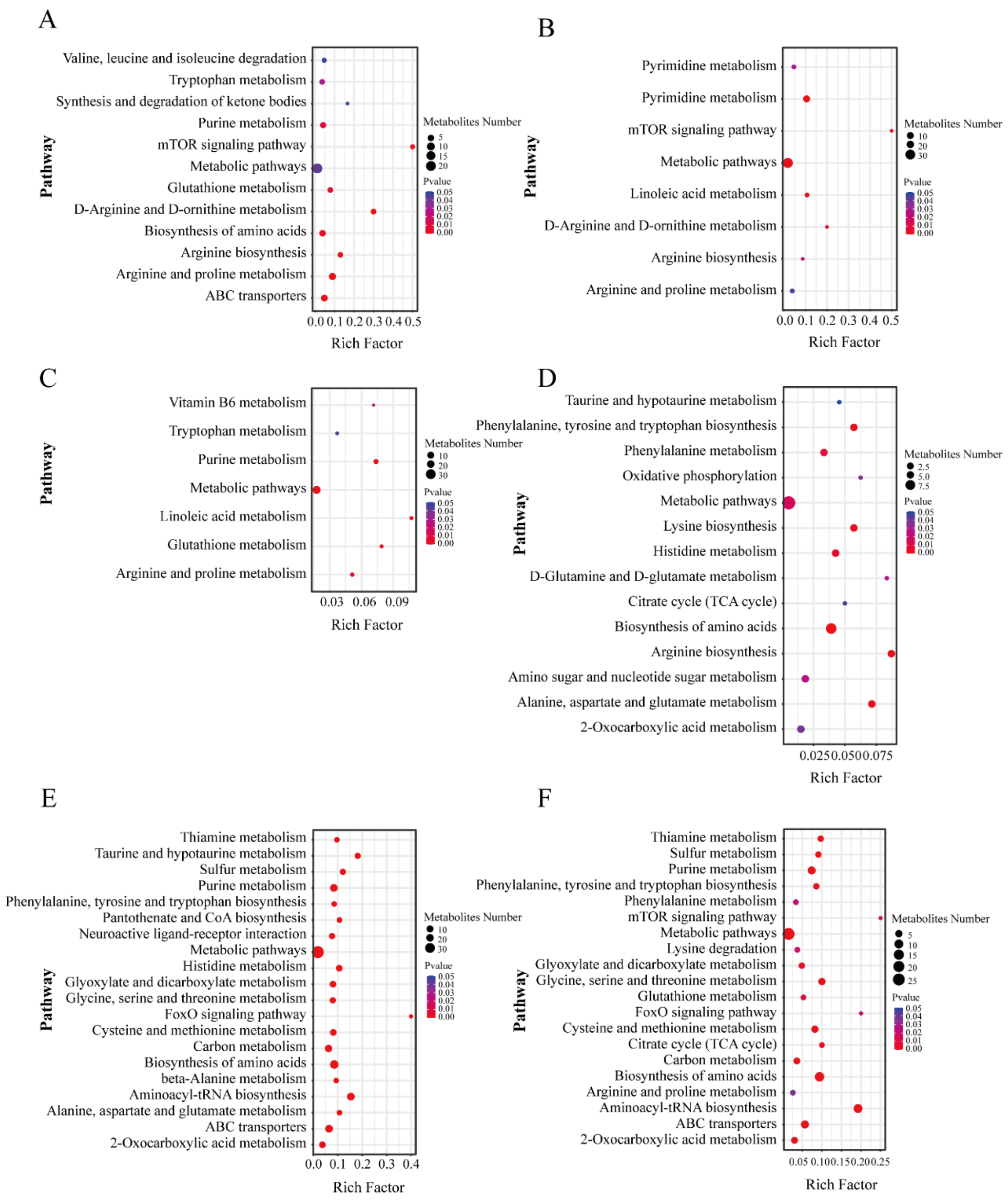

Figure S5.

KEGG enrichment analysis of differential metabolites in different ion scan modes. (A) L vs. H in ESI^+ mode. (B) L vs. H in ESI^- mode. (C) L vs. M in ESI^+ mode. (D) L vs. M in ESI^- mode. (E) M vs. H in ESI^+ mode. (F) M vs. H in ESI^- mode. H, high growth performance level; M, medium growth performance level; L, low growth performance level.
