# Supplementary figures and images for: Alterations of the Gut Microbiota and Metabolomics Associated with the Different Growth Performances of Macrobrachium rosenbergii Families
Source: Animals (Basel). 2023 May 4;13(9):1539. doi: 10.3390/ani13091539 (PMC10177557; doi:10.3390/ani13091539)

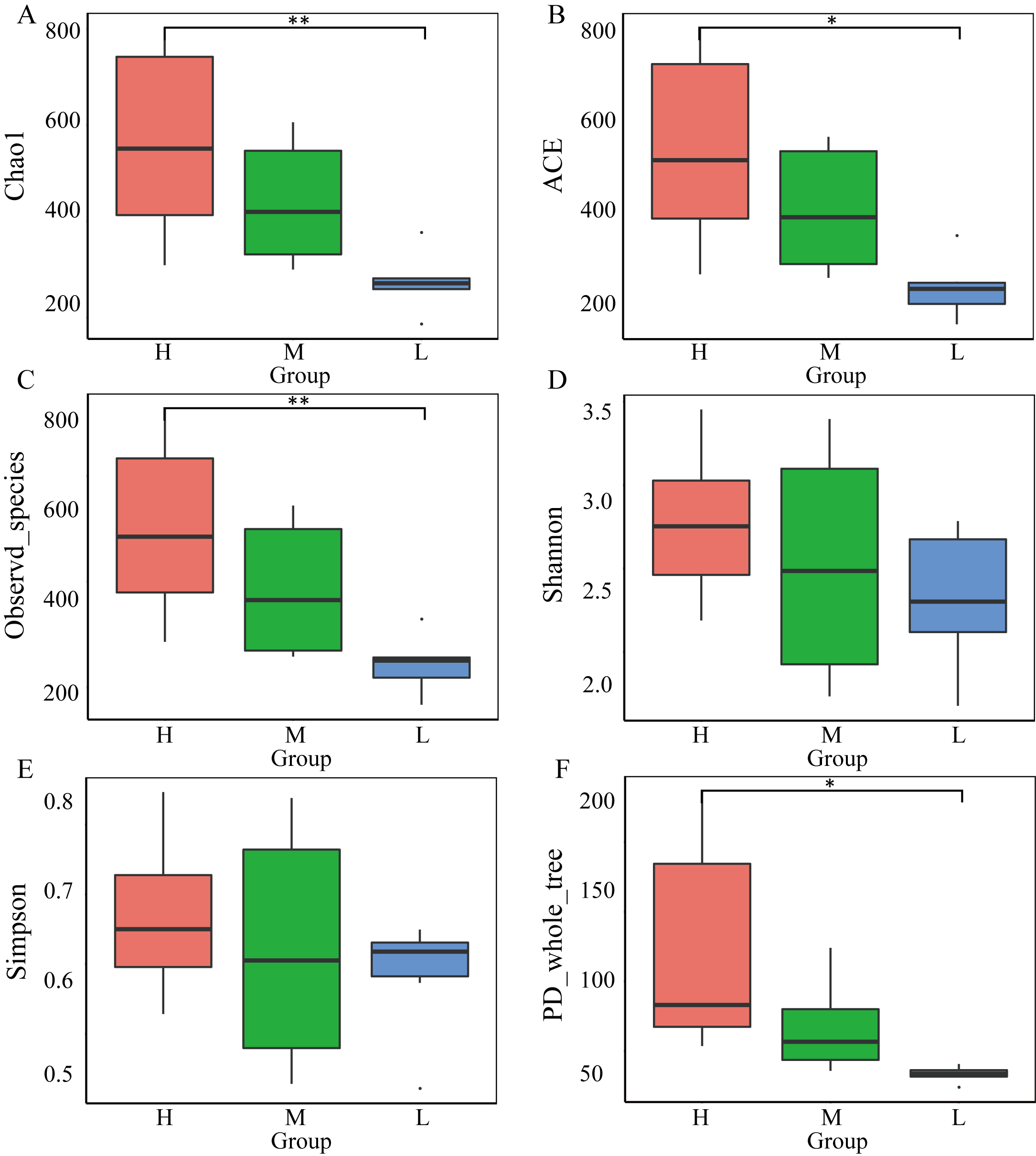

Supplement: Supplementary file 1 [file animals-13-01539-s001.zip › Figure S1.tif]

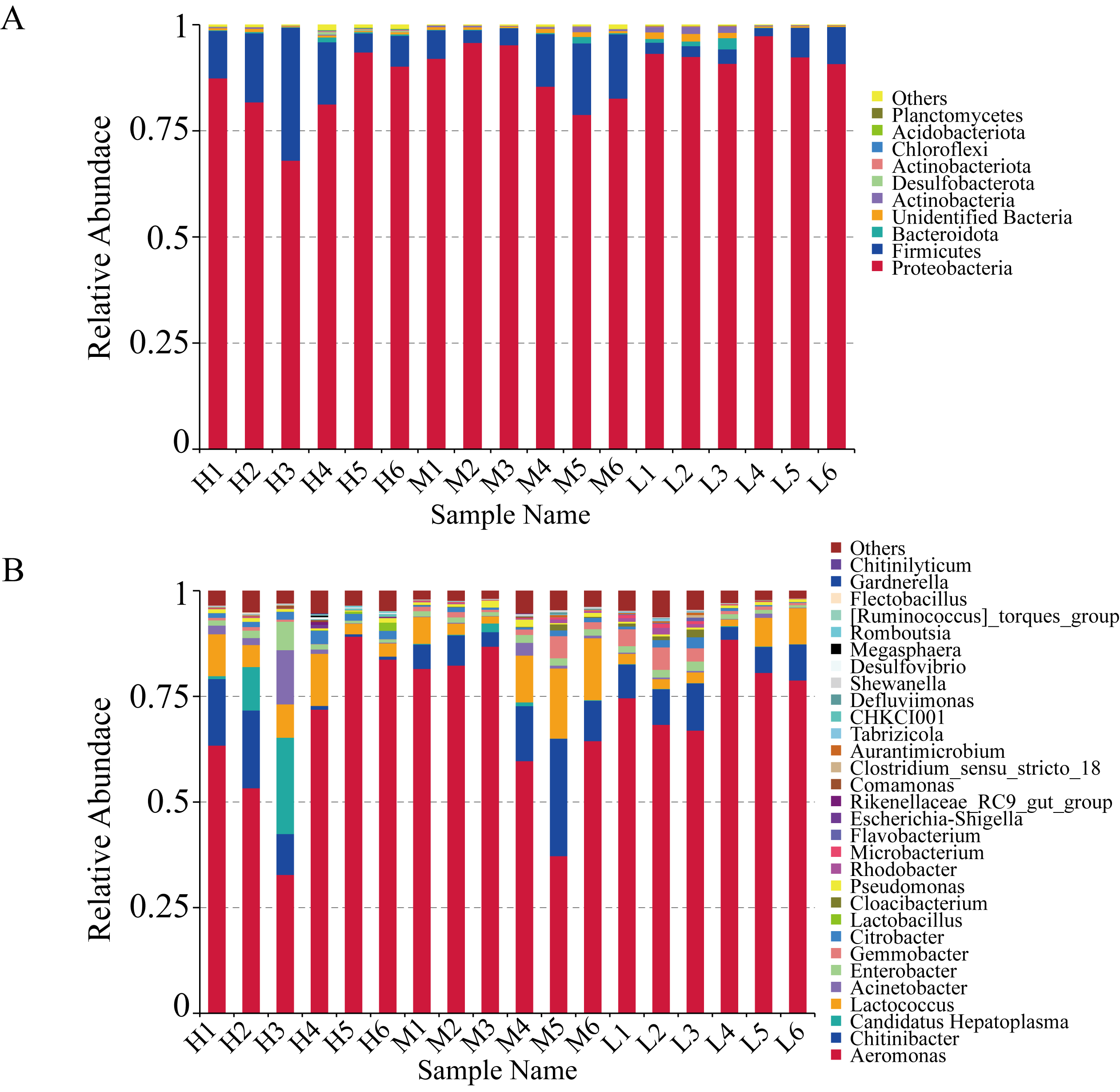

Supplement: Supplementary file 1 [file animals-13-01539-s001.zip › Figure S2.tif]

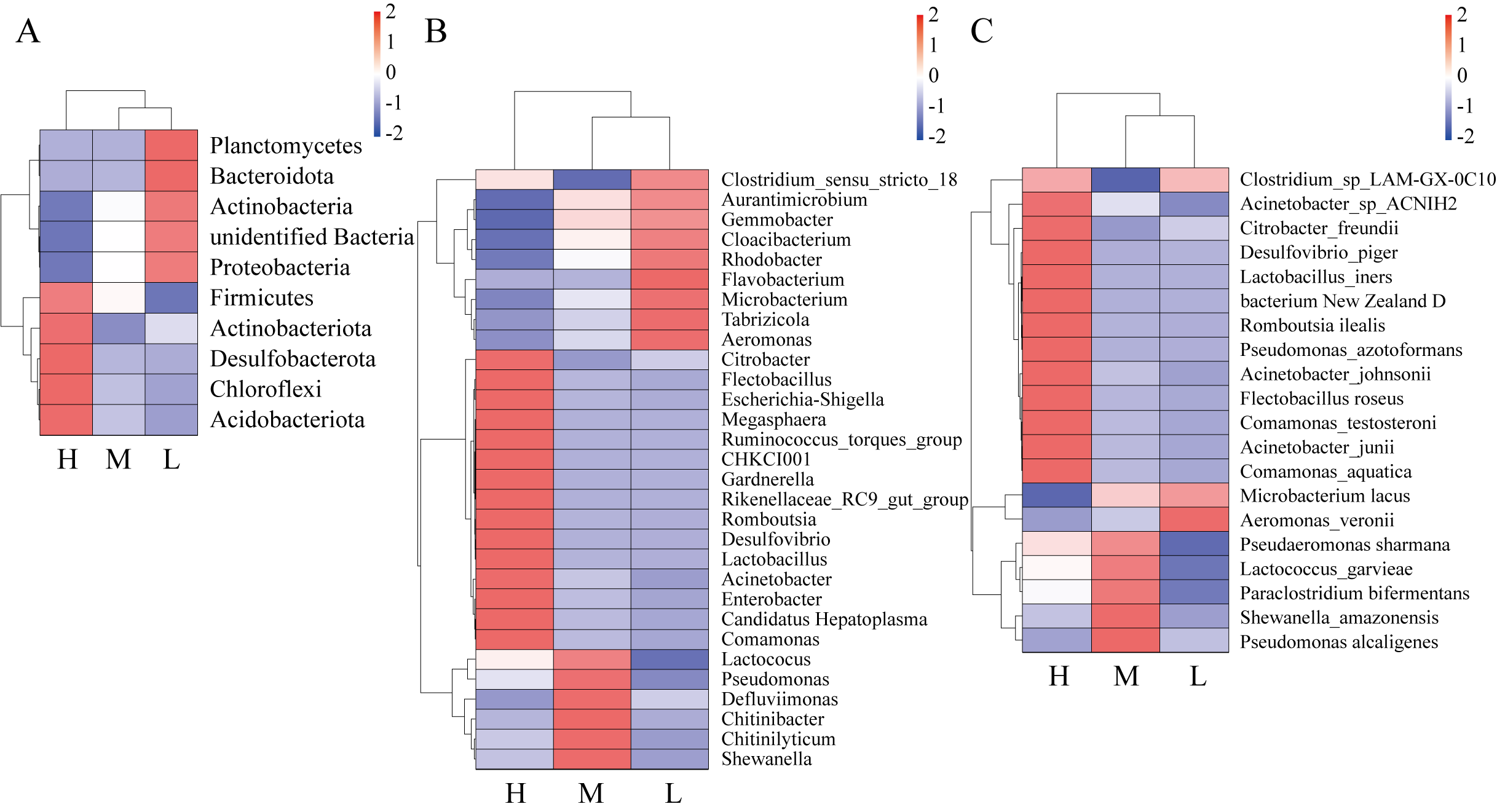

Supplement: Supplementary file 1 [file animals-13-01539-s001.zip › Figure S3.tif]

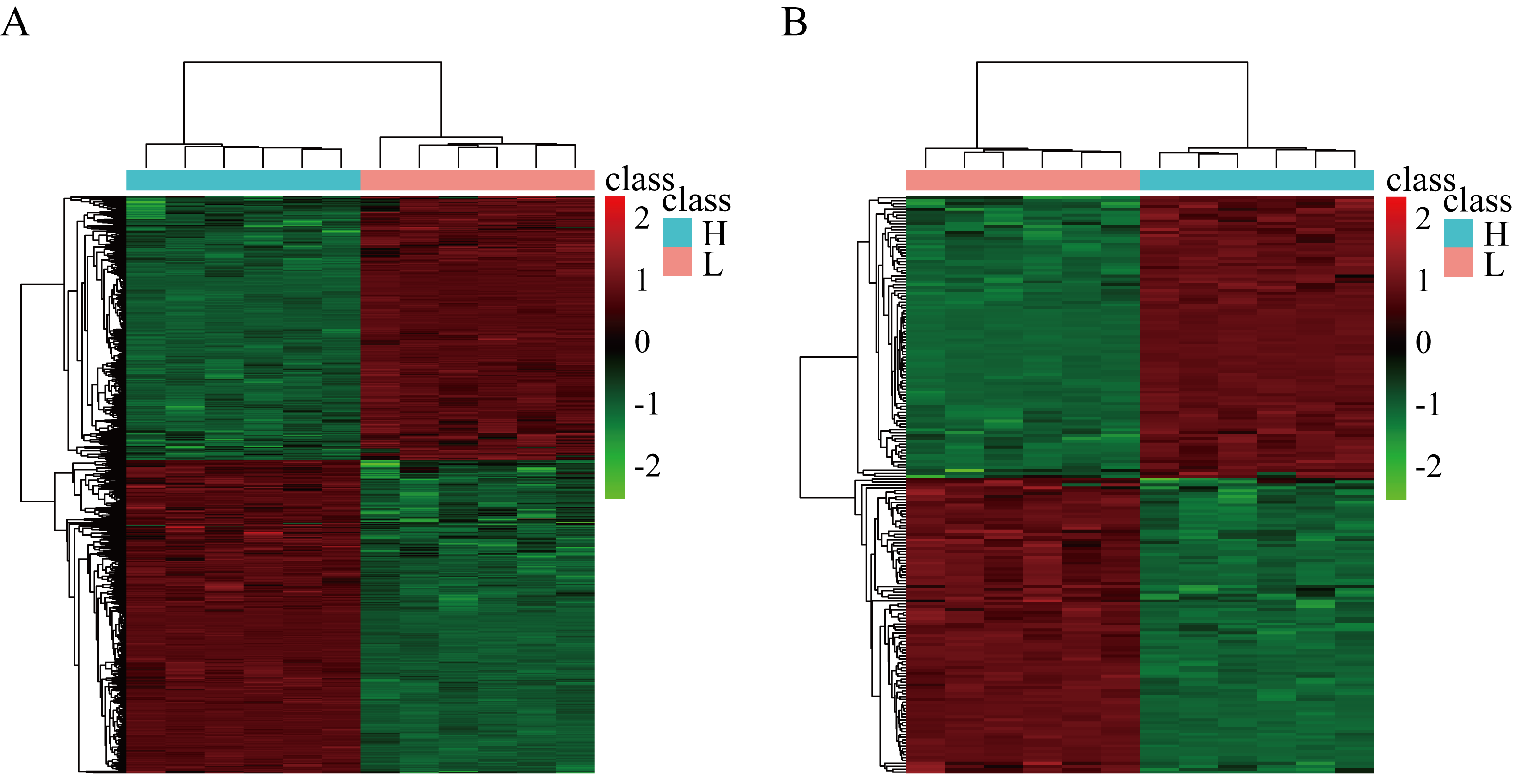

Supplement: Supplementary file 1 [file animals-13-01539-s001.zip › Figure S4.tif]

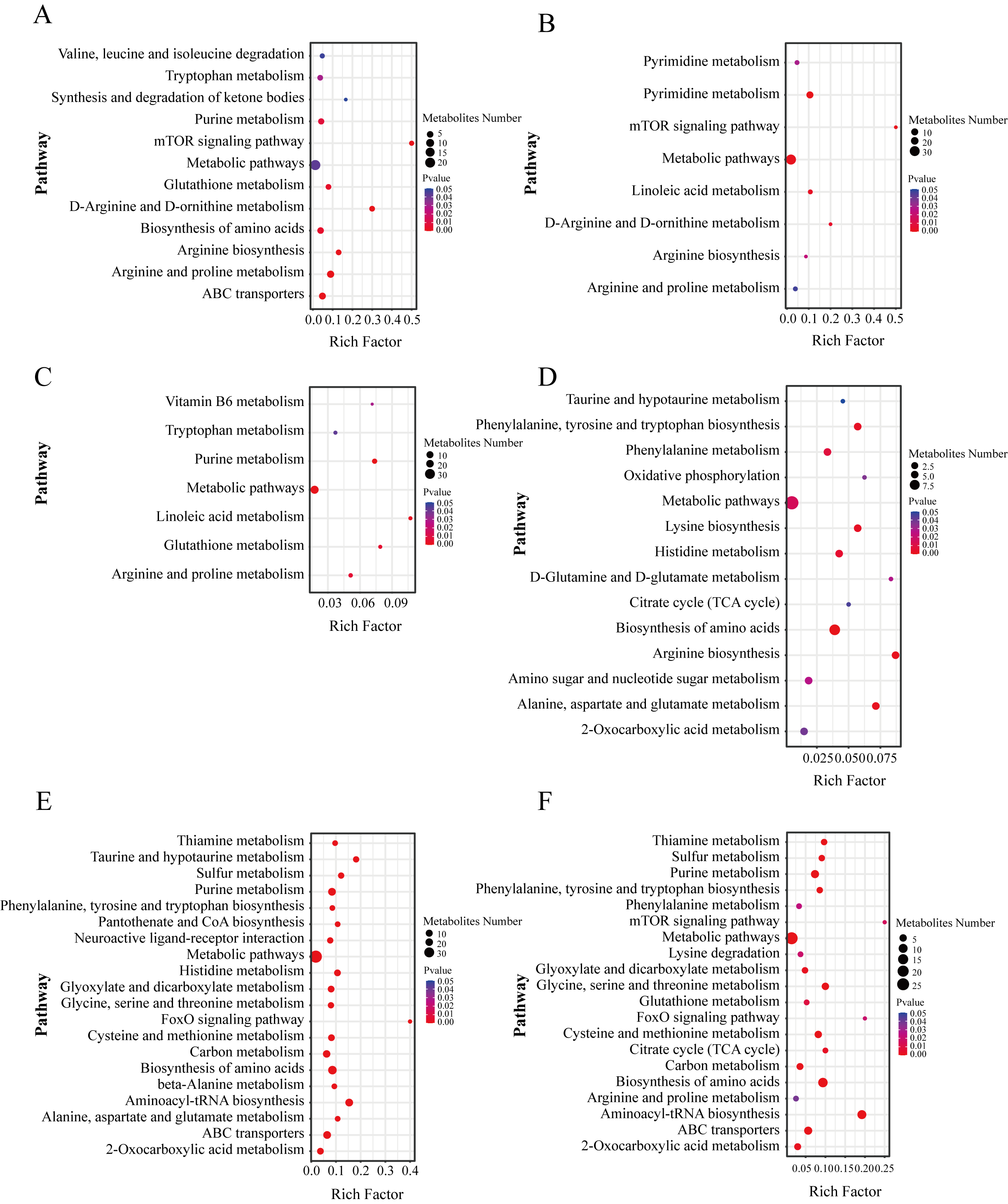

Supplement: Supplementary file 1 [file animals-13-01539-s001.zip › Figure S5.tif]
